# Study on land use and landscape pattern change in the Huaihe River Ecological and economic zone from 2000 to 2020

**DOI:** 10.1016/j.heliyon.2023.e13430

**Published:** 2023-02-27

**Authors:** Mou You, Zeduo Zou, Wei Zhao, Wenwen Zhang, Canfang Fu

**Affiliations:** a College of Geography and Environmental Science, Henan University, Kaifeng 475001, China; b Key Laboratory of Geospatial Technology for the Middle and Lower Yellow River Regions, Ministry of Education, College of Geography and Environmental Science, Henan University, Kaifeng 475001, China

**Keywords:** Land use, Landscape pattern, GRA, Ecological and economic zone, Huaihe river

## Abstract

Exploring the relationship between land use change and landscape patterns can provide a basis for regional ecological management. In this paper, based on remote sensing images of the Huaihe River Ecological and Economic Zone for the years 2000, 2005, 2010, 2015 and 2020, the spatial and temporal evolution patterns of land use in the region were quantitatively described by using the methods of land use shift matrix and landscape pattern analysis. The relationship between land use change and landscape pattern was analyzed with the Grey Relation Analysis (GRA) model. The results show that: (1) the land use of the Huaihe River Ecological and Economic Zone has changed significantly in the past 20 years, with the conversion of arable land into construction and forest lands, in addition to the growth of water areas and a decline in the areas of arable land, grassland and unused land. (2) The landscape pattern fragmentation of each type of land in the study area from 2000 to 2020 fluctuated and decreased, and the landscape connectivity and landscape diversity increased significantly. (3) The GRA model shows that construction, arable and forest lands played the most significant role in the change of landscape pattern of the Huaihe River Ecological and Economic Zone. Countermeasures are proposed to better coordinate and optimize the relationship between spatial development and landscape pattern for the Huaihe River ecological and economic Zone.

## 1 Introduction

Land is an important ecosystem component that guarantees human survival and development [1]. Land use change results from material and energy interactions between the human and ecological environments and shapes landscape patterns [2,3]. Landscape patterns are formed by the spatial arrangement of land use patches of different categories, sizes, and shapes, with clearly distinct spatial and temporal characteristics, and are a concrete expression of landscape heterogeneity [4,5]. At present, land use changes are increasingly accelerating. In the Huaihe River Basin, reducing the impact of land use changes on landscape patterns while achieving high-quality ecological and economic development has become of great concern to the government and academia [6,7]. The Huaihe River Basin, which is located between the Yangtze River basin and the Yellow River basin, is regarded as one of the most promising areas in the middle and east of China. The Huaihe River Ecological Economic Belt covers 221 million mu of arable land, accounting for 11% of the country's total, with a grain production of about 1/6 of the country and a commodity grain accounting for about 1/4 of the country, providing a solid guarantee for national food security.

Land use type change has gradually become a key area of research in the fields of ecology and geography [8], and is mainly studied from the points of view of processes and trends [9,10], influence and mechanisms of human activities [11], and landscape pattern effects and response measures [12,13]. Natural forces and human activities play an important role in shaping landscape patterns [14]. Human activities (e.g., population growth, urban expansion, agricultural mechanization) change the relationship between humans and nature by influencing landscape patterns and accelerating the transformation of natural land into agricultural and construction land, which eventually leads to significant fragmentation of the landscape. Previous studies have mainly focused on the quantitative relationship between regional social and economic aspects and landscape patterns. Scholars have studied the changes in landscape patterns and their driving forces mainly from the following aspects: ① The characteristics of spatial and temporal changes in regional landscape patterns. Through the interpretation of remote sensing image data, the Fragstats software is used to calculate landscape indices such as fragmentation, diversity, and landscape structure, to analyze and monitor regional landscape pattern changes [[15], [16], [17], [18], [19], [20]]. ② To explore the impact of landscape patterns on ecological security. Using techniques such as geographic information systems (GIS) and spatial analysis, the current state of regional ecological security is analyzed in terms of spatial and temporal differentiation [[21], [22], [23], [24], [25]]. ③ Driving mechanisms of spatial and temporal changes in landscape patterns. Remote sensing, GIS, and econometric analysis models are used to explore the changes in landscape patterns and their relationship with the driving forces [[26], [27], [28], [29]]. Scholars have also considered the economic and social factors to study the impact of urbanization development on landscape patterns and the relationship between urbanization and landscape patterns [30]. However, research on the intensity and direction of the impact of urbanization on landscape pattern changes has not been developed. Therefore, this study explores the intensity and direction between land use change and its impact on landscape pattern change through spatial and temporal characteristics to provide a reference for more precise development planning in the Huaihe River Ecological and Economic Zone.

The Huaihe River Ecological and Economic Zone is an important grain production base in central China. Since the plan to promote the development of central China was proposed in 2004, the urban expansion of the Huaihe River Ecological and Economic Zone has accelerated significantly and its land use types have become increasingly diverse, resulting in large changes in its landscape patterns. Therefore, based on remote sensing images of the area, we quantitatively analyzed the spatial and temporal changes in its land use and landscape pattern and applied a Grey Relation Analysis (GRA) to analyze the relationship between land use changes and landscape pattern to provide a reference for regional planning while safeguarding its environment through sustainable development.

## 2 Research methods and data sources

### 2.1 Overview of the study area

**Fig. 1:**
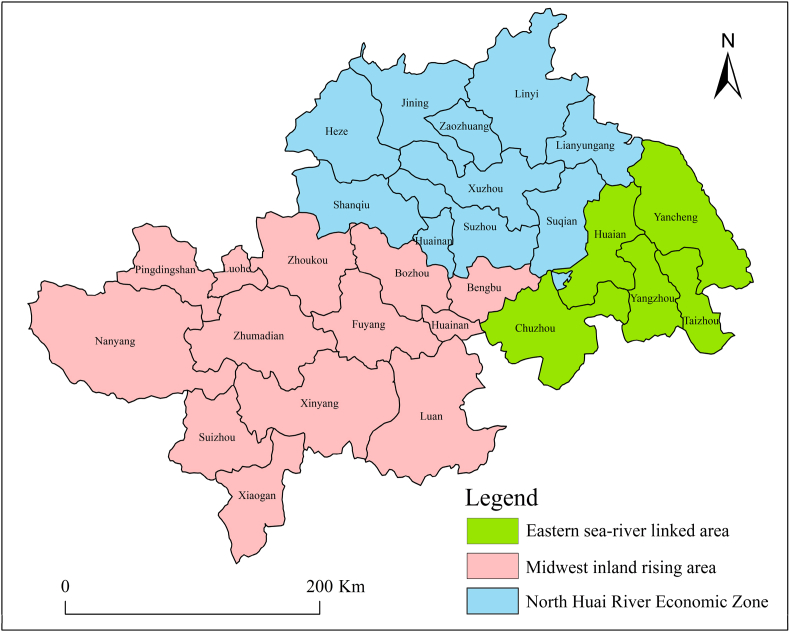
Overview of the study area.

The Huaihe River Ecological and Economic Zone (111°55′–121°25′E, 30°5′–36°36′N) covers a planned area of 243,000 km^2^, starting from Tongbai and Fuyu mountains in the west, bordering the Yellow Sea in the east, divided from the Yangtze River Economic Zone by Dabie Mountain, JiangHuaihe Hills and Tongyang Canal in the south, and adjacent to the Yellow River Economic Zone along the Yellow River South Embankment and Taishan Mountain in the north. It is located in the north-south climate transition zone of China, with a mild climate, rich biodiversity, dense population, abundant human resources, and rapid development of new industrial clusters between the Yangtze and the Yellow rivers economic zones. It is also one of the regions in the middle and eastern part of our country with the most potential for development. We must build on the existing foundation, thoroughly implement the new development concepts, promote the formation of a new pattern of modernization in harmony between man and nature, and create an ecological economic belt with clear water, green land and blue sky [44]. In 2018, the Huaihe River Ecological and Economic Zone was elevated to a national strategy, and this move was a major opportunity to achieve high-quality development in the region. According to the spatial pattern of “one zone, three zones, four axes and more points” proposed in the Huaihe River Ecological and Economic Zone Development Plan, the 28 cities in the Huaihe River Ecological and Economic Zone, including the eastern sea-river linked area, the north Huaihe Economic Zone and the midwest inland rising area, are used as case areas (Fig. 1). The research on land use planning and landscape pattern of the Huaihe River Ecological Economic Belt can be divided into "One Belt (Green Development Belt of the main stream of the Huaihe River)", three areas (Eastern sea-river linked area, North Huai River Economic Zone and Midwest inland rising area), four axes (Linyi – Lianyungang – Suqian – Huaian – Yancheng -Yangzhou - Taizhou development axis, Luohe - Zhumadian -Xinyang development axis, Heze - Shangqiu – Bozhou – Fuyang - Liu'an Development Axis, Jining – Zaozhuang – Xuzhou - Huebei – Suzhou - Bengbu – Huainan - Chuzhou development axis), and multi-point (cities other than the central cities) spatial pattern construction proposed theoretical suggestions.

### 2.2 Research methods

#### 2.2.1 Land use transfer matrix

The land use transfer matrix is an important research method for studying dynamic land use change. It represents the specific characteristics of the transfer structure and transfer direction between each land use type at the beginning and end of the study. The transfer matrix reflects the source, destination, transfer from and to land use type and type composition of landscape type transformation [31]. With the help of the ArcGIS10.3 software, the data of three periods before and after the land use change were calculated by spatial overlay analysis to generate the land use transfer matrix, through which the transformation data between land use types can be obtained and the evolution process of different types of land use can be analyzed [32]. The generalized form of the land use transfer matrix is:

(1) $$S_{ij}=\left[\begin{array}{ccc} S_{11} & \begin{array}{cc} S_{12} & \ldots \end{array} & S_{1n}\\ \begin{array}{c} S_{21}\\ \vdots \end{array} & \begin{array}{cc} \begin{array}{c} S_{22}\\ \vdots \end{array} & \begin{array}{c} \ldots \\ \vdots \end{array} \end{array} & \begin{array}{c} S_{2n}\\ \vdots \end{array}\\ S_{n1} & \begin{array}{cc} S_{n2} & \ldots \end{array} & S_{nn} \end{array}\right]$$

where S is the area. $S_{ij}$ represents the area of the transition from the i-th land use type in the initial stage to the j-th land use type in the final stage; n is the number of land use types. In the transition matrix, the rows represent the *i*-th land use type in the initial stage and the columns represent the *j*-th land use type in the final stage.

#### 2.2.2 Landscape pattern index analysis method

The landscape pattern index can condense the landscape pattern information and quantitatively reflect its characteristics such as pattern composition and spatial configuration [33]. In this study, we used the Fragstats4.2 software to calculate the Huaihe River Ecological and Economic Zone landscape pattern index and selected four indicators in the type level index: Patch Density (PD), Largest Path Index (LPI), Area-weighted Mean Patch Fractal Dimension (FRAC_AM), and Patch Cohesion Index (COHESION). In the landscape level index, we selected the Number of Patches (NP), Landscape Shape Index (LSI), Contagion Index (CONTAG), Shannon's Diversity Index (SHDI) and Shannon's Evenness Index (SHEI), and other five representative indicators [42,43] (Table 1).

**Table 1:** The landscape pattern index.

Indicator	Code	Variable	Description
Level of type	(PD)	Patch Density	Reflects the density of the patch
	(LPI)	Largest Path Index	Reflect the degree of patch concentration and landscape dominant type
	(FRAC_AM)	Area-weighted Mean Patch Fractal Dimension	Reflects the complexity of patch and landscape pattern at a certain observation scale
	(COHESION)	Patch Cohesion Index	Reflect the aggregation and dispersion of patches in landscape
Level of landscape	(NP)	Number of Patches	Equal to the total number of plaques of a certain type
	(LSI)	Landscape Shape Index	Reflects the changing shape of the landscape. The larger the value, the more complex the shape
	(CONTAG)	Contagion Index	Describe the agglomeration degree and spread trend of different patch types in landscape
	(SHDI)	Shannon's Diversity Index	Reflect landscape heterogeneity, that is, describe the uneven distribution of different patch types in the landscape
	(SHEI)	Shannon's Evenness Index	A measure of patch diversity, which is determined by the distribution of the proportion of different land-use types in a landscape.

#### 2.2.3 Grey correlation ratio (GRA) model

Grey correlation analysis is a statistical analysis technique, mainly used to analyze the closeness of the relationship between mother factors and sub-factors in the system, so as to judge the main factors and secondary factors causing the development and change of the system. It is a quantitative comparative analysis method for the dynamic development of the system [39]. Compared with traditional mathematical statistics analysis methods (such as regression analysis, variance analysis, principal component analysis, etc.), grey correlation analysis is equally applicable to sample size and sample irregularity. Moreover, the calculation amount is small, so there will be no inconsistency between quantitative results and quantitative analysis results, which makes up for the limitations of systematic analysis by mathematical statistics method. Therefore, grey relational analysis is used in this study [40].

Land use change is a major factor contributing to landscape pattern change. Understanding how different land use types contribute to landscape pattern change can lead to better landscape pattern planning. To study the relationship between the land use and landscape pattern change, it is necessary to understand both the direction and magnitude of land use change and to identify the type and change of landscape patterns. For this purpose, this paper assumed that landscape pattern change is the result of the influence of land use change. The relationship between land use change and landscape pattern was analyzed using grey correlation ratio (GRA), in which land use type is taken as a sub-factor and landscape pattern change is taken as a parent factor. GRA studies the study area as a whole in the process of system development. If the trend of the change in two factors is consistent, there is a high degree of synchronous change, which means that the degree of correlation between the two is high, and vice versa. The specific calculation steps are as follows [7]:

In the first step, set the system characteristic behavior sequence as $S_{i}\left(t\right)=\left\{S_{i}\left(1\right),S_{i}\left(2\right),\cdots ,S_{i}\left(t\right)\right\}$, the related factor behavior sequence as $S_{0}\left(t\right)=\left\{S_{0}\left(1\right),S_{0}\left(2\right),\cdots ,S_{0}\left(t\right)\right\}$, and their dimensionless characteristics as ${S_{i}}^{\prime }\;\left(t\right)=\left\{{S^{\prime }}_{i}\left(1\right),{S^{\prime }}_{i}\left(2\right),\cdots ,{S^{\prime }}_{i}\left(t\right)\right\}$; the related factor behavior sequence as ${S_{0}}^{\prime }\left(t\right)=\left\{{S^{\prime }}_{01}\left(1\right),{S^{\prime }}_{02}\left(2\right),\cdots ,{S^{\prime }}_{0t}\left(t\right)\right\}$, i = 1,2,3, …,n, t = 1,2,3, …, m, where n is the number of system characteristic behavior sequence, and m is the number of indicators.

In the second step, the absolute difference between ${S_{i}}^{\prime }\left(t\right)$ and ${S_{0}}^{\prime }\left(t\right)$ as well as the maximum and minimum values are calculated after dimensionless processing of the original sequence. The calculation formula is (2), (3), (4):

(2) $${△}_{i}\left(t\right)=\left|S_{0}^{\prime }\left(t\right)-S_{i}^{\prime }\left(t\right)\right|$$

(3) $${△}_{\min}=\min_{i}\min_{t}\left|S_{0}^{\prime }\left(t\right)-S_{i}^{\prime }\left(t\right)\right|$$

(4) $${△}_{\max}=\max_{i}\max_{t}\left|S_{0}^{\prime }\left(t\right)-S_{i}^{\prime }\left(t\right)\right|$$

The third step is to calculate the correlation coefficient $\gamma_{i}\left(t\right)$ as (5):

(5) $$\gamma_{i}\left(t\right)=\frac{\Delta_{\min}+{\rho \Delta }_{\max}}{\Delta_{i}\left(t\right)+{\rho \Delta }_{\max}}$$

where ρ is the resolution coefficient, ρ∈[0,1]; the larger the value of ρ, the smaller the resolution, and usually ρ is taken as 0.5.

In the fourth step, the correlation degree $\delta_{i}$ is found as (6):

(6) $$\delta_{i}=\frac{1}{m}\sum_{t=1}^{m}\gamma_{i}\left(t\right)$$

Where t=1,2,3,⋯,m*.* $\delta_{i}$ represents the grey correlation of $S_{i}^{\prime }\left(t\right)$ for $S_{0}^{\prime }\left(t\right)$.

### 2.3 Data source and processing

#### 2.3.1 Data source

In this paper, five Landsat TM remote sensing images of 2000, 2005, 2010, 2015 and 2020 were obtained from the geospatial data cloud(http://www.gscloud.cn/search). The ENVI5.1 software was used to pre-process the remote sensing images of each year, referring to the standard of China's Land Use Status Classification (GB/T21010-2017), combining visual interpretation with Google Earth software with actual field surveys, and using the maximum likelihood method to perform a supervised classification of the study area [34].

**Fig. 2:**
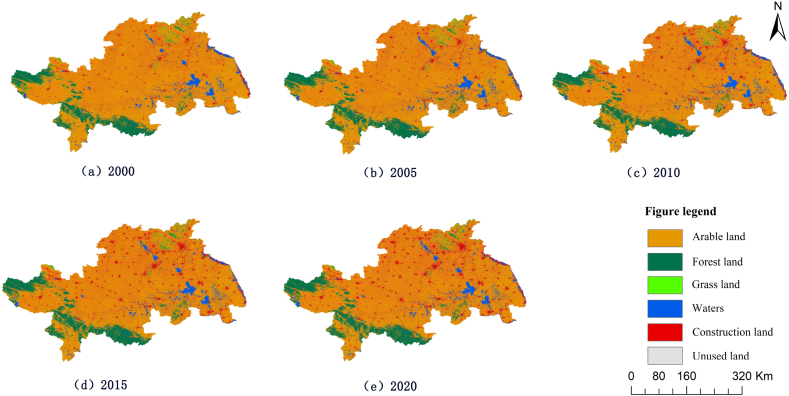
Spatial distribution of land types in the Huaihe River Ecological Economic Zone from 2000 to 2020.

The land use types were classified into six types: arable land, forest land, grassland, water area, construction land and unused land. Finally, the images were post-processed using ArcGIS10.3 to obtain a five-phase land use type map of the Huaihe River Ecological and Economic Zone (Fig. 2).

#### 2.3.2 Accuracy evaluation methods

At present, the construction of confusion matrix is usually used as one of the important accuracy evaluation methods for the post-processing of ground cover classification, and specifically the level of classification accuracy is judged by the overall accuracy (OA) or Kappa statistic.

Generally, the confusion matrix is constructed in the form of a table, and the larger the value of the elements on the diagonal of the confusion matrix, the higher the reliability of the classification results, and vice versa, the more serious the classification errors. Based on the overall accuracy (OA) and Kappa coefficient in the confusion matrix construction are defined as follows [37].

(7) $$OA=\frac{\sum_{i=1}^{n}x_{ii}}{N}$$

(8) $$K=\frac{N\sum_{i=1}^{n}x_{ii}-\sum_{i=1}^{n}\left(x_{i+}x_{+i}\right)}{N^{2}-\sum_{i=1}^{n}\left(x_{i+}x_{+i}\right)}$$

Where: OA is the overall accuracy; N is the total number of sample points used for precision evaluation; n is the total number of columns in the confusion matrix; $x_{ii}$ is the number of sample points in row i and column i of the confusion matrix; $x_{i+}$ and $x_{+i}$ are the total number of sample points in row i and column i, respectively; K is the Kappa coefficient.

When the value of K is 0, it indicates only random consistency, when the value of K is 1, it indicates full consistency between people or measurement instruments, and if it is between 0 and 1 there is only a rough distinction. Table 2 shows the interpretation of the differentiated classification of Kappa between 0 and 1 and the corresponding high or low consistency [38].

**Table 2:** *K*-factor interpretation.

*K*	Degree of consistency	*K*	Degree of consistency
<0.02	Poor	0.41–0.60	Moderate
0.02–0.20	Slight	0.61–0.80	Substantial
0.21–0.40	Fair	0.81–1.00	Almost Perfect

## 3 Results

### 3.1 Analysis of land use change in the Huaihe River Ecological and economic zone

#### 3.1.1 Accuracy evaluation

The results of remote sensing land use mapping of the Huaihe River Ecological and Economic Zone for five periods of 2000, 2005, 2010, 2015 and 2020 were evaluated in terms of accuracy. For the six primary land use types, a confusion matrix was established based on the remote sensing classification results and Google Earth sampling information to calculate the overall accuracy and Kappa coefficient, and the summarized calculation results are shown in Table 3. The results show that the kappa values for all five periods are greater than 0.81 and the OA values are greater than 90%. These high fitting values indicate that the accuracy of the land classification simulation is high and the land use remote sensing mapping shows good consistency [41].

**Table 3:** Accuracy of land use remote sensing mapping.

Precision Type	2000	2005	2010	2015	2020
OA	90.61%	93.90%	92.43%	95.65%	94.39%
Kappa	0.8832	0.9180	0.8917	0.9342	0.9110

#### 3.1.2 The characteristics of land use structure change

The land uses of the study area from 2000 to 2020 are shown in Table 4.

**Table 4:** The area and proportion of various types of land use in the Huaihe River Ecological and Economic Zone from 2000 to 2020.

Land use type	2000		2005		2010		2015		2020	
	area/km^2^	proportion/%	area/km^2^	propor tion/%	area/km^2^	propor tion/%	area/km^2^	propor tion/%	area/km^2^	propor tion/%
cultivated land	218391.29	76.43	214006.59	74.90	210866.99	73.80	205259.43	71.84	204089.91	71.43
woodland	26167.73	9.16	27311.93	9.56	27183.08	9.51	28629.33	10.02	28008.95	9.80
grass land	2016.18	0.71	1645.44	0.58	1476.14	0.52	1277.33	0.45	904.31	0.32
waters	10311.20	3.61	11862.86	4.15	11891.20	4.16	11687.14	4.09	11231.38	3.93
construction land	28388.20	9.93	30463.48	10.66	33877.38	11.86	38445.40	13.45	41064.68	14.37
Unused land	446.91	0.16	431.20	0.15	426.71	0.15	422.87	0.15	422.27	0.15

Combined with Table 1 and Fig. 2, it can be seen that cultivated land and construction land constitute the main body of land use in the Huaihe River Ecological and Economic Zone from 2000 to 2020, accounting for 86.7% of the total area in the study area, which is the main type of land use in the study area. It proves that in the context of rapid urbanization, agricultural production is still an important production method in the Huaihe River Ecological and Economic Zone. HuaiheIn descending order of area share, they are forest land, water, grassland and unused land. In the past 20 years, the area of cultivated land and grassland in the Huaihe River Ecological and Economic Zone has been decreasing continuously. Woodland area fluctuated greatly, showing a trend of "decreasing - increasing - decreasing”. The continuous increase of construction land area indicates that the urbanization process of the Huaihe River Ecological and Economic Zone is accelerating. The water area showed a fluctuating trend of "increasing - decreasing - increasing”. The continuous decrease of unused land area indicates that the local relevant departments are paying increasing attention to the effective use of land resources.

#### 3.1.3 Land use transfer analysis

The land use transfer matrix from 2000 to 2010 and 2010–2020 reveals the transfer relationship between each category (Table 5 and Table 6).

**Table 5:** Land use area transfer matrix of the Huaihe River Ecological and Economic Zone from 2000 to 2010.

Year	Type	2010						
		cultivated land	woodland	grass land	waters	construction land	Unused land	Total transfers out
2005	cultivated land	–	2229.59	254.24	2075.03	5789.87	0.61	10349.34
	woodland	1268.35	–	28.53	2.79	23.42	0.00	1323.09
	grass land	636.66	101.23	–	13.50	73.62	4.45	829.46
	waters	847.63	7.48	2.92	–	254.88	0.32	1113.23
	construction land	69.84	0.14	0.52	592.27	–	0.03	662.81
	Unused land	2.56	–	3.22	9.64	10.20	–	25.62
	Total transfers in	2825.04	2338.44	289.43	2693.23	6151.99	5.41	–

**Table 6:** Land use area transfer matrix of the Huaihe River Ecological and Economic Zone from 2010 to 2020.

Year	Type	2020						
		cultivated land	woodland	grass land	waters	construction land	Unused land	Total transfers out
2010	cultivated land	–	2387.70	257.11	1388.37	6947.63	1.20	10982.00
	woodland	1736.18	–	8.85	3.12	26.26	0.05	1774.46
	grass land	583.58	205.39	–	6.98	41.70	1.33	838.98
	waters	1849.47	7.22	0.39	–	458.75	0.46	2316.29
	construction land	34.16	0.02	0.06	256.99	–	0.05	291.28
	Unused land	1.53	–	0.75	1.01	4.24	–	7.53
	Total transfers in	4204.92	2600.33	267.15	1656.47	7478.57	3.09	–

In general, the land use transfer characteristics of the Huaihe River Ecological and Economic Zone in the past 20 years were: arable land was mainly transferred out, construction land and forest land were mainly transferred in, and the total area of water, grassland and unused land was transferred to a smaller extent.

### 3.2 Analysis of landscape pattern change of the Huaihe River Ecological and economic zone

#### 3.2.1 Type level

**Fig. 3:**
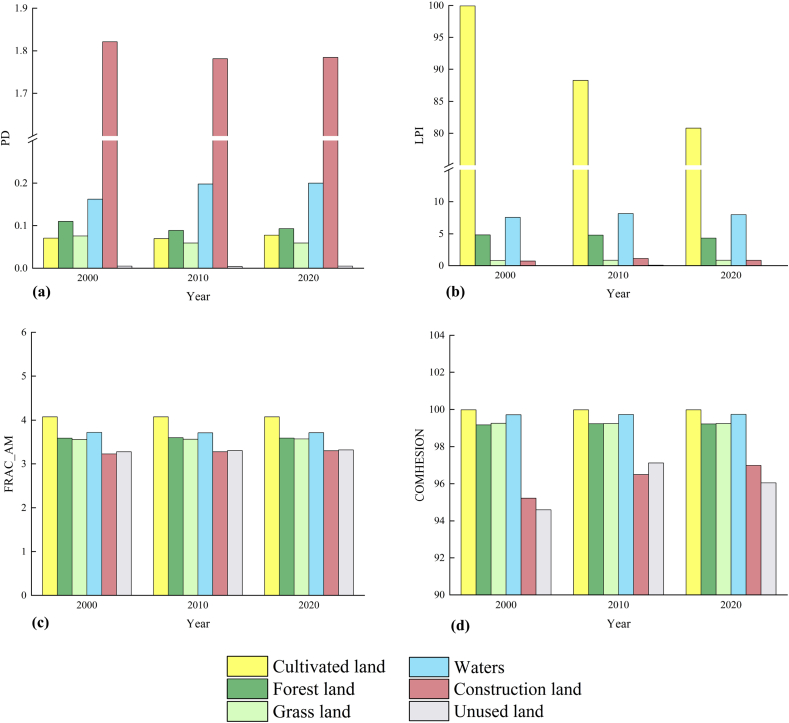
Changes of horizontal landscape indices of different landscape types in Huaihe River Ecological Economic Zone from 2000 to 2020.

The analysis of the type-level change characteristics of the Huaihe River Ecological and Economic zone (Table 7, Fig. 3), Fig. 3(a)shows that from 2000 to 2020, the patch density of unused land did not change significantly, while that of woodland, grassland, and construction decreased and that of cultivated land and waters increased. This indicates that the fragmentation of arable land and waters increased and the landscape dynamics stabilized. It can be seen from Fig. 3 (a) that in recent 20 years, the maximum patch index of cultivated land and water was larger, indicating that the dominant landscape in the study area was cultivated land and water; after 2000, the maximum patch index of cultivated land gradually decreased and the maximum patch index of water fluctuated, while the area of cultivated land decreased and the area of water fluctuated, indicating that the landscape dominance of cultivated land in the Huaihe River Ecological and Economic Zone decreased and that of water increased; the landscape dominance of construction land increased. The maximum patch index of construction land increased, indicating that its landscape dominance also increased. According to Fig. 3(c), the area-weighted average fractal dimension of cultivated land is the largest, indicating that the shape distribution of cultivated land is irregular; the average fractal dimension of construction land and unused land is smaller, indicating that the two types of land are greatly affected by human activities. According to the Patch Cohesion Index of various types of land use (Fig. 3(d))， the aggregation index of arable land and forest land as a whole was greater than 99% due to their larger areas; the aggregation index of water area, which was characterized by strong connectivity, was also greater than 99%; the aggregation index of construction land gradually increased, indicating that the area of construction land grew in a concentrated and contiguous manner as urbanization accelerated.

**Table 7:** Landscape index of landscape type level in the Huaihe River Ecological and Economic Zone from 2000 to 2020.

landscape type	Year	PD	LPI	FRAC-AM	COMHESION
cultivated land	2000	0.071	99.930	4.074	99.98
	2010	0.070	88.250	4.074	99.98
	2020	0.078	80.817	4.075	99.98
woodland	2000	0.110	4.834	3.587	99.18
	2010	0.089	4.805	3.596	99.24
	2020	0.093	4.338	3.588	99.23
grass land	2000	0.076	0.802	3.556	99.26
	2010	0.059	0.835	3.563	99.25
	2020	0.059	0.835	3.569	99.25
waters	2000	0.162	7.544	3.716	99.72
	2010	0.198	8.129	3.707	99.73
	2020	0.200	7.965	3.710	99.74
construction land	2000	1.821	0.718	3.223	95.23
	2010	1.781	1.112	3.280	96.49
	2020	1.784	0.834	3.303	96.98
Unused land	2000	0.005	0.008	3.279	94.59
	2010	0.004	0.083	3.306	97.11
	2020	0.005	0.017	3.320	96.05

#### 3.2.2 Landscape level

**Fig. 4:**
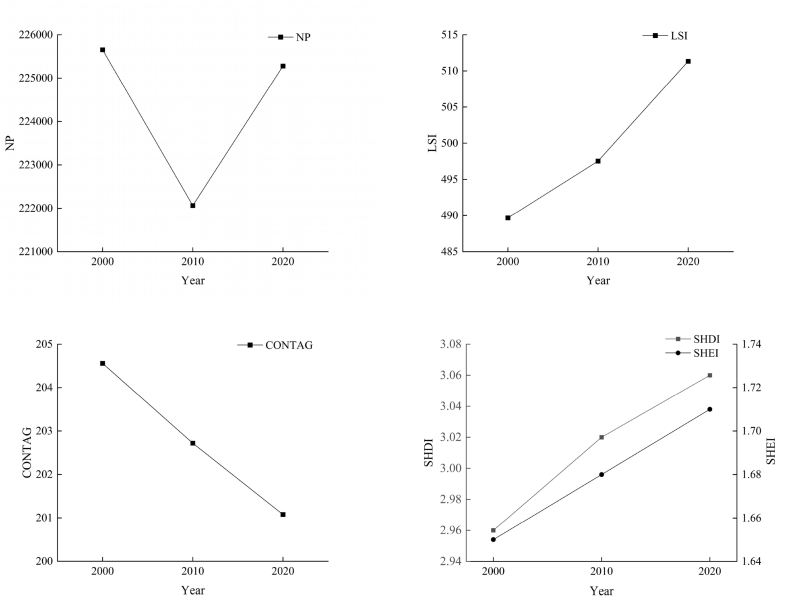
Changes of different landscape level indices in the Huaihe River Ecological and Economic Zone from 2000 to 2020.

The analysis of the landscape level change characteristics shows that the number of patches decreased and then increased between 2000 and 2020 (Fig. 4); the overall number of patches in the Huaihe River Ecological and Economic Zone decreased by 375 patches during the study period (−0.16%). The number of patches decreased from 2000 to 2010. The overall fragmentation of the landscape was relatively small during this period, and the number of patches increased after 2010, indicating an increase in human activities. The landscape shape index gradually increased from 2000 to 2010, indicating an increase in the complexity of the land type shape and irregularity of the patches, reflecting the growing fragmentation and dispersion of the landscape patches in the study area after 2000. The spreading index continuously decrease between 2000 and 2020, indicating that the connectivity of the dominant patches in the Huaihe River Ecological and Economic Zone decreased as the landscape fragmentation increased. The Shannon diversity index and Shannon evenness index, reflecting the degree of heterogeneity of the landscape, gradually increased between 2000 and 2020, indicating that the distribution of each type of land in the Huaihe River Ecological and Economic Zone was homogeneous and the diversity of the landscape increased.

### 3.3 The relationship between land use change and landscape patterns

The SPSS software was used to analyze the magnitude of grey correlation of the six influencing factors of landscape pattern (Table 8). The top three PD-correlation rankings were for arable land (0.93) > construction land (0.92) > forest land (0.86); the top three LPI-correlation rankings were for arable land (0.75) > construction land (0.73) > forest land (0.56); the top three FRAC-AM correlation rankings were for construction land (0.82) > forest land (0.81) >cultivated land (0.80); the top three COMHESION-correlations were construction land (0.83) > forest land (0.80) > cultivated land (0.79); the top three NP-correlations were construction land (0.84) > cultivated land (0.81) > forest land (0.79); the top three LSI- correlations were construction land (0.92) > construction land (0.75)>cultivated land (0.73); the top three CONTAG-correlations were construction land (0.88)>cultivated land (0.85)>forest land (0.75); the top three SHDI&SHEI-correlations were forest land (0.90)>construction land (0.76)>cultivated land (0.74).

**Table 8:** Grey relational statistics table.

	cultivated land	woodland	grass land	waters	construction land	unused land
PD	0.93	0.86	0.62	0.78	0.92	0.64
LPI	0.75	0.56	0.44	0.54	0.73	0.33
FRAC-AM	0.80	0.81	0.35	0.65	0.82	0.39
COMHESION	0.79	0.80	0.36	0.68	0.83	0.36
NP	0.81	0.79	0.33	0.62	0.84	0.38
LSI	0.73	0.92	0.33	0.68	0.75	0.43
CONTAG	0.85	0.75	0.33	0.62	0.88	0.37
SHDI	0.74	0.90	0.33	0.69	0.76	0.42
SHEI	0.74	0.90	0.33	0.69	0.76	0.42

As a whole, there was an evenly distributed strong influence of the six influencing factors on the landscape pattern. The explanatory power of construction land, cultivated land and forest land on each landscape index were always in the top three, indicating that there was a close relationship among these land use types in the Huaihe River Ecological and Economic Zone and that they played the most significant role in the change of the regional landscape pattern.

## 4 Discussion

### 4.1 Influencing factors of landscape pattern change

The results of the integrated landscape pattern response to land use change found that changes in cropland, forest land and construction land drove the spatial heterogeneity of the landscape pattern. The strongest driving effect was the expansion of construction land, which led to the conversion of cropland into construction land. This pattern implies that the intensity of human activities influences land use change, landscape pattern fragmentation, and ecosystem change. This study further sorted out the effects of cropland, forest land, and construction land on landscape pattern changes and provides references for how to reduce the effects of land use changes on landscape patterns in the Huaihe River Ecological and Economic Zone.

Cropland. Arable land is an important indicator of the level of regional food security. During the study period, the Huaihe River Ecological and Economic Zone implemented afforestation policies such as "returning farmland to forest”, which directly promoted changes in cultivated land and forested land and resulted in a significant increase in the transfer of cultivated land to forested landscape types. At the same time, the demand of economic development in the study area has increased the intensity of demand for construction land, resulting in a rapid decline in the area of arable land.

Construction land. Construction land is an important measure of the level of regional economic development. During the study period, increase in GDP in the study area was achieved through a continuous expansion of construction land to grow new cities, which led to a decrease in the area of arable land and other land types that increased the heterogeneity and fragmentation of the landscape pattern of the entire study area.

Woodland. Woodland is an important measure of regional ecological service functions. During the study period, the Huaihe River Ecological and Economic Zone cut down European and American species of poplar trees to protect the wetland area, thus leading to a decline in woodland area. At the same time, the study area belongs to the alluvial plain and lacks topographic factors that could constrain the development of construction land, resulting in its rapid expansion and increasing the degree of landscape fragmentation and the complexity of patch shape, creating obstacles to maintaining the structural stability of regional ecosystems.

Overall, rapid population growth, economic development, and industrial level in the Huaihe River Ecological and Economic Zone were the main factors leading to the changes in land use and landscape patterns; land use patterns and regional planning and policies were also one of the important reasons for the changes in landscape patterns. At the same time, the rapid urbanization of the region has encouraged investors to invest more in industrial and town development and infrastructure; therefore, there has been an increase in the number of population movements and the establishment of new urban areas, which has led to dramatic land use changes. Similarly, the results of [35] showed that the construction of development zones, transportation networks, and other projects had a significant impact on land use changes, leading to the transformation of most land types [7]. Therefore, with the expansion of construction land, the regional landscape was gradually replaced by construction land, destroying the region's natural ecosystem and fragmenting the landscape pattern.

### 4.2 Enlightenment for regional landscape pattern planning

As an important ecological and economic zone in China, the urban built-up areas in the study area have been booming in recent years, and urban construction land in suburban and distant suburban areas has been expanding, causing drastic impacts on the existing land use pattern [36]. Studies have shown that there is spatial heterogeneity in the impact of land use change on the landscape patterns of the Huaihe River Ecological and Economic Zone. Using GRA to explore the correlation between land use change and landscape pattern can provide a basis for decision-making for spatial high-quality development planning. Therefore, when formulating the high-quality development planning of Huaihe River Ecological and Economic Zone, the following measures need to be taken to avoid the destruction of the landscape pattern, which affects the region's sustainable development. Firstly, the optimal regulation of land use planning should focus on the protection of watershed, forest land and arable land to maintain and strengthen the continuity of the landscape pattern of the region as a whole and reduce the degree of fragmentation of the landscape pattern. At the same time, the protection of green areas in urban and suburban areas should be strengthened to restore landscape diversity. The land use type in the region during the period 2000–2020 was dominated by the growth of construction land and the gradual decrease in natural or semi-natural grassland area. Despite the construction of urban green areas such as parks, the negative impact of land use changes on the landscape pattern has been increasing during the study period. Therefore, policymakers should focus on protecting natural or semi-natural green areas, building more green landscapes, and increasing the diversity of urban landscapes. Second, the ecological red line of the Huaihe River Ecological and Economic Zone should be delineated to strictly control the scale of construction land. The expansion of construction land during the period 2000–2020 has encroached on a large amount of arable land and grassland, resulting in an increasing fragmentation of the landscape pattern. Therefore, it is necessary to implement the concept of compact city, limit the growth of construction land and increase the intensity of protection of arable land and grassland in the process of building new zones in the Huaihe River Ecological and Economic Zone. In response to the increased fragmentation and landscape shape complexity in the study area, policymakers should focus on protecting woodland landscapes in mountainous areas, as well as woodland, grassland and arable landscapes in semi-mountainous areas. Favoring the spatial connection of natural landscape patches through large-scale woodlands and selecting watersheds with high landscape ecological can help promote the sustainable development of the entire ecosystem of the region.

Based on land use transfer matrix and landscape pattern analysis, this paper studied the change characteristics of land use and landscape pattern in the Huaihe River Ecological Economic Zone from 2000 to 2020, and then discussed the relationship between land use change and landscape pattern. Taking the river basin as the research area, combined with natural factors, social and economic factors, from the time scale and spatial scale of the overall and macro grasp and analysis. Land use change is a very complex process involving many factors. Due to the limitation of the accuracy of remote sensing data and the integrity of the socio-economic statistical data of provinces and cities in the Huaihe River Basin, the basic data in this paper are not comprehensive and accurate, and the temporal and spatial resolution are low. At the same time, the expression forms and description ways of landscape pattern change are diverse, and there is no complete system. In future studies, more factors should be added to build a more complete factor system, construct a variety of landscape pattern expression forms, and reflect the relationship between land use change and landscape pattern from different scales.

## 5 Conclusion

We quantitatively analyzed the characteristics of land use and landscape pattern changes in the Huaihe River Ecological and Economic Zone during 2000–2020 and then explored the relationship between land use changes and landscape patterns. First, Landsat remote sensing images were classified to identify the spatial and temporal land use change characteristics; then, the landscape index was used to study the change characteristics of the landscape pattern in the area; finally, the relationship between land use change and landscape pattern was analyzed by using the grey correlation degree method. The results show that.

(1) There were significant land use changes in the Huaihe River Ecological and Economic Zone during 2000–2020. Dynamic changes showed a decline of arable land, grassland and unused land, and the expansion of construction land, forest land and water area; the shift of arable land was the most obvious, mainly due to construction land and forest land; forest land, water area and unused land also shifted to other land types, mainly forest land, grassland and water area are shifted out to arable land and unused land is shifted out to construction land, respectively. Overall, the largest amount of land use area change in the Huaihe River Ecological and Economic Zone in the past 20 years was for arable land and construction land, and the overall characteristics of land use transfer are: arable land was mainly transferred out, construction land and forest land are mainly transferred in, and the transferred area of watershed, grassland and unused land is smaller.
(2) During 2000–2020, land use changes in the Huaihe River Ecological and Economic Zone led to significant changes in landscape patterns. From the type level, the landscape dominance of cultivated land decreased and that of waters increased; the landscape of construction land spatially converged; in comparison, construction land and unused land were influenced more by human activities; waters showed strong connectivity, and the area of construction land grew in a concentrated and continuous manner. From the viewpoint of landscape level, the fragmentation of landscape pattern of each type of land decreased, landscape connectivity increased, and landscape diversity increased during the study period.
(3) In this study, the GRA model was used to analyze the influence mechanism of landscape pattern changes in the Huaihe River Ecological and Economic Zone, and it was found that construction land, arable land and forest land played the most significant role in the landscape pattern changes in the Huaihe River Ecological and Economic Zone. However, some limitations remain in our approach. On the one hand, the analysis was conducted from the aspect of land use, without considering the influence of economic, social and ecological factors on the landscape pattern change. On the other hand, this study analyzed the influence of landscape pattern change from the policy perspective, but no quantitative analysis was conducted. Future research can adopt a multi-objective optimization approach to optimize landscape patterns from the perspective of multiple scenarios and provide a reference for future landscape pattern change and sustainable development of the Huaihe River Ecological and Economic Zone.

## Author contribution statement

Mou You: Conceived and designed the experiments; Analyzed and interpreted the data; Wrote the paper.

Zou Zeduo: Conceived and designed the experiments; Analyzed and interpreted the data.

Zhao Wei: Performed the experiments; Analyzed and interpreted the data.

Zhang Wenwen: Performed the experiments; Contributed reagents, materials, analysis tools or data.

Fu Canfang: Analyzed and interpreted the data; Contributed reagents, materials, analysis tools or data.

## Funding statement

This work was supported by National Natural Science Foundation of China [41271144].

## Data availability statement

Data associated with this study has been deposited at http://www.gscloud.cn/search

## Declaration of interest’s statement

The authors declare no conflict of interest.
